# FAMILY EXPERIENCE IN USING TELEPRESENCE ROBOTS WITH RESIDENTS IN LONG-TERM CARE

**DOI:** 10.1093/geroni/igae098.3551

**Published:** 2024-12-31

**Authors:** Lillian Hung, Grace Hu, Joey Wong, Haopu (Lily) Ren, Jim Mann, Lily Wong

**Affiliations:** University of British Columbia, Vancouver, British Columbia, Canada; Columbia University, New York, New York, United States; University of British Columbia, Vancouver, British Columbia, Canada; University of British Columbia, Vancouver, British Columbia, Canada; University of British Columbia, Vancouver, British Columbia, Canada; University of British Columbia, Vancouver, British Columbia, Canada

## Abstract

Informal care, most often provided by family or friends, remains a hidden and underrecognized undertaking within long-term care (LTC) settings. As care recipients transition into LTC, informal care partners experience a parallel shift in their caregiving roles: transitioning from primary care partner to visitor. Many care partners report this transition to be difficult, as they must surrender an unpredictable level of control and involvement over their loved one’s care. The COVID-19 pandemic intensified this experience by introducing new challenges: individual health concerns, fluctuating care home protocols, and isolating government policies. Telepresence robots are emerging as a tool that can ease the care partner transition. The main aim of this study is to examine the experiences of care partners who used telepresence robots with loved ones living in LTC settings, during the COVID-19 pandemic. This study took place between May 2021 and August 2023 in five urban LTC homes located in British Columbia, Canada. A total of 20 care partners were recruited through purposive sampling. The care partners participated in semi-structured interviews, in-person or virtually, and thematic analysis was employed to identify four key themes characterizing their experiences using the robot: 1) Decreases care partner burden, 2) Facilitates care partner-staff relationship, 3) Creates relational autonomy, and 4) Expands the scope of what is possible. Findings capture the ability of the robot to enhance the caregiving experience for informal care partners. Further research on the sustainability of robot implementation amongst diverse geographic regions and care home compositions is needed.

